# The Essential Role of Type I Interferons in Differentiation and Activation of Tumor-Associated Neutrophils

**DOI:** 10.3389/fimmu.2016.00629

**Published:** 2016-12-21

**Authors:** Ekaterina Pylaeva, Stephan Lang, Jadwiga Jablonska

**Affiliations:** ^1^Translational Oncology, Department of Otolaryngology, University Hospital Essen, Essen, Germany

**Keywords:** type I interferons, neutrophils, tumor, inflammation, neutrophil polarization

## Abstract

Type I interferons (IFNs) were first characterized in the process of viral interference. However, since then, IFNs are found to be involved in a wide range of biological processes. In the mouse, type I IFNs comprise a large family of cytokines. At least 12 IFN-α and one IFN-β can be found and they all signal through the same receptor (IFNAR). A hierarchy of expression has been established for type I IFNs, where IFN-β is induced first and it activates in a paracrine and autocrine fashion a cascade of other type I IFNs. Besides its importance in the induction of the IFN cascade, IFN-β is also constitutively expressed in low amounts under normal non-inflammatory conditions, thus facilitating “primed” state of the immune system. In the context of cancer, type I IFNs show strong antitumor function as they play a key role in mounting antitumor immune responses through the modulation of neutrophil differentiation, activation, and migration. Owing to their plasticity, neutrophils play diverse roles during cancer development and metastasis since they possess both tumor-promoting (N2) and tumor-limiting (N1) properties. Notably, the differentiation into antitumor phenotype is strongly supported by type I IFNs. It could also be shown that these cytokines are critical for the suppression of neutrophil migration into tumor and metastasis site by regulating chemokine receptors, e.g., CXCR2 on these cells and by influencing their longevity. Type I IFNs limit the life span of neutrophils by influencing both, the extrinsic as well as the intrinsic apoptosis pathways. Such antitumor neutrophils efficiently suppress the pro-angiogenic factors expression, e.g., vascular endothelial growth factor and matrix metallopeptidase 9. This in turn restricts tumor vascularization and growth. Thus, type I IFNs appear to be the part of the natural tumor surveillance mechanism. Here we provide an up to date review of how type I IFNs influence the pro- and antitumor properties of neutrophils. Understanding these mechanisms is particularly important from a therapeutic point of view.

## Introduction

The significance of type I interferons (IFNs) in cancer immune surveillance is well established by now. These cytokines were first characterized late in the 1950s as cytokines with antiviral activity (1). In the mouse, type I IFNs comprise a large family of cytokines with at least 12 IFN-α, IFN-β, IFN-ε and IFN-κ (2, 3). All of them signal *via* a common receptor IFNAR, and they induce the expression of several 100 IFN-inducible genes and have a broad range of biological functions (2). Within the type I IFNs, IFN-α and IFN-β are best characterized. Importantly, a hierarchy of expression has been shown to exist for these cytokines (4, 5), where IFN-β is induced first. When it binds to IFNAR, IFN-β in a paracrine and autocrine fashion triggers a cascade of type I IFNs, including IFN-α and IFN-β. The only exception to this rule are plasmacytoid dendritic cells (pDCs), which can start immediately with the secretion of IFN-α (6). Besides, its importance for the induction of the IFN cascade, IFN-β is also constitutively expressed in low amounts under normal non-inflammatory conditions (7). This was clearly demonstrated by non-invasive imaging using the new luciferase reporter mouse, but also by *ex vivo* determination of the enzymatic activity of luciferase in various tissues (4). The reason for such constitutive expression of IFN-β might be the priming of the immune system to persist in a pre-activated state that guarantees a faster and stronger type I IFNs response when necessary. Notably, using luciferase reporter mouse, it could be demonstrated that growing tumors induce type I IFNs expression mainly in tumor-infiltrating dendritic cells (DCs) (8).

Besides their role in antiviral and antimicrobial responses, type I IFNs shape innate and adaptive immunity (9), influence the maintenance of cellular homeostasis (10), hematopoiesis (11), and lymphocyte development (12). In addition, type I IFNs show strong antitumor activity (13) and are involved in cancer immunoediting (14). The mechanisms of how type I IFNs contribute to the immune surveillance against tumors are not fully understood, notwithstanding their beneficial effects in the cancer therapy (13). In the context of cancer, type I IFNs were found to play a key role in supporting host immune responses through the activation of multiple immune cells, e.g., T-cells, natural killer (NK) cells, DCs, and macrophages. In recent years, it has become apparent that type I IFNs affect also neutrophil activation and promote antitumor functions of these cells.

The inflammation has been recently associated with increased susceptibility for cancer (15). Consequently, neutrophils as a central component of this process play an essential role in inflammation-driven tumorigenesis. Moreover, neutrophils represent an independent prognostic marker in a broad variety of neoplasias. In the past, these cells were viewed as solely dedicated to phagocytosis and the production of reactive oxygen species (ROS). Now, they are recognized for an extreme versatility with regard to function (16, 17) and play manifold roles during tumor development (8, 18). Neutrophils affect primary tumor growth by influencing its angiogenesis (18), but also by direct killing of tumor cells (8). Moreover, neutrophils can facilitate the spread of tumor cells to distant organs in a process called metastasis (19, 20). Neutrophils are apparently controlled by factors produced by the primary tumor and are responsible for the preference of metastasizing tumor cells to certain organs. Type I IFNs have a substantial influence on this process (20).

The tumor microenvironment represents a special niche that is extremely influencing infiltrating immune cells. The concept of immune cell polarization was described initially for macrophages (antitumor M1/pro-tumor M2), but recently polarization of neutrophils is getting growing attention. Neutrophils appear to have contradicting phenotypes in the tumor microenvironment, i.e., tumor promoting (N2) or inhibiting (N1) (16), depending on the cytokine milieu in the tumor. Cytokines that are known to control neutrophil polarization are type I IFNs, driving neutrophil differentiation into N1 antitumor state (8). Of note, strict classification into N1 or N2 phenotypes is certainly an oversimplification. More likely, these two immune phenotypes spot the end points of a continuum of functional states exhibited by neutrophils in tumor milieu, which can be regulated by the environmental cues. Here, we provide an up to date review of how type I IFNs influence the pro- and antitumor properties of neutrophils.

## Tumor Microenvironment and the Phenotype of Neutrophils

Tumor cells, as well as infiltrating immune cells, produce wide range of cytokines, chemokines, and growth factors. This leads to the activation and recruitment of other immune cells, such as neutrophils, monocytes, and lymphocytes. Moreover, the tumor microenvironment plays a significant role in the differentiation and functional properties of such cells. In the early steps of tumorigenesis, the infiltration of immune cells into the tumor tissue serves as one of the tumor killing mechanisms and provides protection against tumor progression. When this line of defense is insufficient and tumor escapes the immune response, the balance shifts to suppressive anti-inflammatory microenvironment. This effect was initially described and widely studied for macrophages and their polarization into antitumor M1 and pro-tumor M2 (21–26).

Neutrophils were previously thought to be terminally differentiated, short lived myeloid cells. Recent studies, however, confirmed that tumor-associated neutrophils (TANs) can also exhibit antitumor N1 or pro-tumor N2 properties, similarly to macrophages (16). The different role of such neutrophil phenotypes in tumor progression and their influence on the prognosis of the disease were assessed. Some studies revealed strong antitumor properties of neutrophils (27), including antibody-dependent or direct cytotoxicity (28) mediated by ROS release (29) and production of neutrophil extracellular traps (NETs) (8). Moreover, neutrophils potentiate antitumor immune responses by the recruitment of other immune cells to the tumor site (30). Recently, the role of neutrophils as possible antigen-presenting cells (APCs) was suggested. These cells were shown to modulate activation of CD4^+^ and CD8^+^ T cells *via* expression of costimulatory molecules like CD86, ICAM-1, OX40L, and 4-1BBL (31–33).

At the same time, tumor-supporting activities of neutrophils were revealed, demonstrating the role of these cells as efficient inhibitors of host immunosurveillance. Moreover, neutrophils were shown to stimulate tumor angiogenesis *via* secretion of vascular endothelial growth factor (VEGF) and matrix metallopeptidase 9 (MMP9) (18). This leads to the better vascularization of the primary tumor and its growth. Of note, not only primary tumor growth is maintained by neutrophils, but also the formation of metastases can be enhanced by these cells. Pro-tumor neutrophils upregulate the expression of pro-metastatic proteins, e.g., Bv8, S100A8, and S100A9, but also VEGF and MMP9 in pre-metastatic lungs of IFN-deficient mice. This phenomenon, together with the enhanced infiltration of lungs by these cells, leads to improved metastatic load in IFN-deficient mice (20). N2 neutrophils are characterized with immature nucleus shape and reduced tumor cell killing capacity (34); they were also shown to recruit regulatory T cells in tumors by expression of CCL17 (35). Accordingly, in clinical studies, the percentage of neutrophils in blood and neutrophil/lymphocyte ratio was shown to be negative predictors of patient outcome in different types of cancer (36, 37).

Although two functionally different types of neutrophils were described, clear markers allowing distinguishing pro- and antitumor neutrophils are still not available. Nevertheless, factors that determine the phenotype shift are in general similar to those for macrophages (16). Type I IFNs are considered as N1-promoting cytokines and transforming growth factor beta (TGF-β) has been suggested to be N2 inducer (38). TGF-β is a well-known immunosuppressive cytokine, expressed also in tumors, which circulating form was shown to correlate with tumor progression (39). The functional antagonism between type I IFNs and TGF-β is observed not only for neutrophils but also was shown for human peripheral blood mononuclear cells (40) and it could be due to the antagonisms between signaling pathways of these cytokines. Smad2 and Smad3, the downstream molecules of the TGF-β signal transduction pathway, were shown to negatively regulate LPS-induced macrophage activation by suppressing multiple steps in the IFN-β/STAT1 pathway, including the inhibition STAT1 transcriptional activity (41). Similar results were shown for myeloid cells of the central nervous system (CNS), where TGF-β impaired the ability of such cells to acquire a resolving anti-inflammatory phenotype *via* downregulation of IFN regulatory factor 7 (IRF7) (42). Thus, TGF-β may potentially influence neutrophil type I IFN-dependent functions and polarization by modulation of STAT1 pathway. Nevertheless, the regulation of type I IFN and TGF-β pathways seems to be more complicated since a positive crosstalk between IFN-α and TGF-β signaling was observed in preneoplastic rat liver, resulting in activation of both; STAT1 and Smad2/3 pathways (43).

Deficiency in endogenous type I IFN signaling seems to play a significant role in the switch of immune response from antitumor to pro-tumor one. Moreover, there is an evidence of changing phenotype of TANs with a shift to pro-tumorigenic properties during tumor progression (44), which can be explained with continuous fluctuation of cytokines and chemokines.

Of note, an alternative concept of anti-inflammatory low-density neutrophils (LDNs) and pro-inflammatory high-density neutrophils (HDNs) in tumor situation emerged recently (45). Due to the literature, HDNs represent a homogenous population of mature segmented neutrophils, while LDN population consists of both immature (banded/MDSC) and mature neutrophils. HDNs are characterized as cells with high cytotoxicity against tumors while LDNs have no cytotoxicity, representing the pro-tumor neutrophils subset (45). Yet, there is no comprehensive data showing the influence of type I IFNs on the LDL/HDL balance in the tumor-bearing hosts. The current knowledge on the heterogeneous populations of mature and immature neutrophils, including LDNs, low-density granulocytes (LDGs), granulocytic myeloid-derived suppressor cells (G-MDSCs), and immunosuppressive neutrophils is summarized recently by Scapini et al. (46).

## Endogenous Type I IFN Signaling in Neutrophils

The receptor of type I IFNs (IFNAR) belongs to the family of type II cytokine receptors that trigger the activation of the JAK-STAT pathway. Ligand that binds to the receptor activates the cascade of phosphorylation of JAK molecules, which in turn phosphorylate tyrosine residues on the receptor chains leading to the subsequent STAT protein phosphorylation and activation. IFNAR primarily utilizes JAK1 with some accessory role for JAK2 and TYK2, and activates STAT1, STAT2, or STAT3 pathways. Phosphorylated STAT proteins undergo dimerization and shuttle to the nucleus where they bind promoter regions and regulate gene transcription (47). Type II cytokine receptors are involved in a number of neutrophil function including regulation of survival, differentiation, and activation (48–50). Different expression of mediating proteins and the regulation of intracellular signal transduction pathways determine the final effect of type I IFNs under certain conditions (51). STAT1/STAT3 functional imbalance with the shift to STAT3 activation and following antiapoptotic protein expression is known to be crucial for tumorigenesis (52). In this case, IFN-β was shown to suppress cancer growth and metastasis rate through inhibition of STAT3 signaling in tumor cells (53).

In the context of cancer, type I IFNs show strong antitumor properties. IFN gene therapy was associated with sustained local production of IFN-β that efficiently suppressed tumor growth in prostate and bladder cancer as well as melanoma, renal cell carcinoma, and colon carcinoma (54–56). This effect was ascribed to the induction of tumor cell apoptosis (57) and the inhibition of tumor angiogenesis due to decreased VEGF expression in different tumors (58, 59).

A new line of studies devoted to the effects of IFNs in tumor conditions was induced by the growing evidence that the immune system plays a significant role in the regulation of oncogenesis. Type I IFNs were shown to stimulate antitumor immune responses *via* several mechanisms reviewed by Parker et al., including enhancement of immune recognition of tumor cells by upregulation of major histocompatibility complex (MHC) class I and tumor antigen expression on tumor cell surface, increasing NK cytotoxicity, and switching macrophage phenotype from M2 to M1 (60). Moreover, antigen-presenting properties of DCs were shown to be improved, effector T cell proliferation enhanced, and suppressive activity of regulatory T cells reduced by these cytokines.

Neutrophils lately are being recognized as key players that regulate tumorigenesis and metastatic processes, modulation of their differentiation and activation by type I IFNs becomes an important area of research. The absence of endogenous type I IFN signaling results in shift of neutrophil phenotype to tumor-supporting one. Several factors can be responsible for this phenomenon, including genetic peculiarities of molecular signaling pathways (61, 62), maturation state of the neutrophils, and exogenous influence. While the genes for intracellular proteases and other cytotoxic proteins were shown to be expressed at earlier stages of maturation, the genes for proteins responsible for signal transduction from IFNAR, and, therefore, mediating the release of abovementioned cytotoxic factors, are preferentially induced during terminal differentiation (63). Immature state of circulating human neutrophils exposed to granulocyte/macrophage colony-stimulating factor (GM-CSF) *in vitro* was characterized with downregulation of IFN signaling pathway, including IFNAR, IFN-γ receptor as well as JAK1, JAK2, STAT1, and STAT2 (63). Colony-stimulating factors are usually overexpressed in tumor environment. Considering the fact that these factors induce the release of immature neutrophils from the bone marrow (BM) as well as support the immunosuppressive state in circulation (64–66), one can expect a downregulation of IFN-mediated signal transduction pathways and decreased efficiency of antitumor immune responses in the presence of such growth factors. Similar feedback loop was described for VEGF, one of the factors supporting tumor angiogenesis, which is capable to downregulate IFNAR expression (67).

Although type I and type II IFN signaling pathways share common intracellular mediators, e.g., STAT1, STAT2, and STAT3, they are shown to exhibit different regulatory role in tumorigenesis. While IFN-γ is known mainly as an agent regulating tumor cell survival, type I IFNs primarily modulate host immune responses against tumors (68). The exclusive effect of type I IFNs on the host immune system was also confirmed by studies of Wu et al. that could demonstrate enhanced tumor growth in animals lacking IFNAR but able to produce endogenous type I IFNs (20). In this case, tumor growth was similarly enhanced as in IFN-β-deficient animals. The constitutive lack of endogenous IFN-β (*Ifnb1^−/−^* mice) as well as the lack of type I IFN signaling (*Ifnar1^−/−^* mice) leads to increased growth of different types of tumors (B16F10 melanoma, 4T1 mammary carcinoma, LLC carcinoma, and MCA205 fibrosarcoma) (8, 18, 20, 34, 69) and enhanced metastatic processes (20). The strong pro-tumor phenotype of *Ifnb1^−/−^* mice confirms the hypothesis that the expression of all alpha IFNs strongly depends on the previous IFN-β expression. Even if pDCS are indeed able to express alpha IFNs without previous stimulation, the comparable elevated tumor growth in *Ifnb1^−/−^* and *Ifnar1^−/−^* mice demonstrates its irrelevance. Enhanced tumorigenesis in type I IFN-deficient mice is accompanied by strong accumulation of neutrophils in primary tumors. These neutrophils show reduced cytotoxicity, increased pro-angiogenic properties, and are resistant to apoptosis.

## Type I IFNs Influence the Turnover and the Lifespan of Neutrophils in Tumor Environment

Neutrophils for long time were believed to be short-living cells. This was most probably due to *ex vivo* manipulation techniques that limited the lifespan of these cells. Recently, the perspective is changing and there are data demonstrating much longer neutrophil lifespan that can reach up to approximately 10–20 days (70). Neutrophil homeostasis in the organism is maintained through a balance of neutrophil production, release from the BM, and clearance from the circulation (71). The BM serves as a reservoir for neutrophils that can be rapidly mobilized in response to inflammatory stimuli. However, at steady state, only a small fraction of the total BM neutrophil pool is released into circulation (72). In the absence of pro-inflammatory stimuli, neutrophils undergo spontaneous apoptosis and are phagocyted by tissue macrophages (73). Several stimuli can prolong neutrophil survival, including infectious factors associated with bacterial infections (LPS) as well as colony-stimulating factors, e.g., granulocyte colony-stimulating factor, G-CSF (74).

Tumors are known to produce the whole spectrum of colony-stimulating factors (IL-3, G-CSF, and GM-CSF) (64–66, 75) that potentially influence proliferation of progenitor cells, neutrophil release from BM, and prolongation of their lifespan in tissues. Other sources of G-CSF are endothelial cells (76) and neutrophils themselves (34). Type I IFNs were shown to downregulate G-CSF expression on gene and protein level (20, 34). G-CSF is known to be a major regulator of neutrophil development, mobilization, and differentiation. It has been shown to mobilize neutrophils from BM to the blood *via* STAT3 activation and regulating CXCL2/CXCR2 axis (20, 77) as well as to suppress neutrophil apoptosis (78). Furthermore, recently Casbon et al., using a multistage mouse model of breast cancer, could show that tumor-derived G-CSF was responsible for both the development and activity of immunosuppressive neutrophils in cancer (79). Similarly, Spiegel et al. could recently show that G-CSF-induced neutrophils act to promote metastasis in 4T1 lung metastasis model *via* inhibition of NK cell-mediated clearance of intraluminal tumor cells. Moreover, such neutrophils facilitate extravasation of tumor cells into lungs *via* secretion of IL1β and matrix metalloproteinases (80).

Since G-CSF is upregulated in type I IFNs-deficient tumor-bearing mice (34), this phenomenon could be responsible for the observed neutrophil expansion in bloodstream and tumors of such mice (18). Generally, TANs are characterized with prolonged lifespan comparing to other tissue neutrophils (81). Andzinski et al. demonstrated that endogenous type I IFNs influence neutrophil survival and lifespan. In type I IFNs-deficient mice bearing B16F10 melanoma, the neutrophil life span was prolonged due to apoptosis suppression. Neutrophilic granulocytes from such *Ifnb1^−/−^* tumor-bearing mice expressed higher amounts of BCL-xL and showed decreased effector caspase 3 activity as well as inhibited expression of death receptor Fas (34). Fas expression on neutrophils has been shown to be involved in spontaneous extrinsic cell death signaling (82). Even though Fas ligand-induced apoptosis is considered not to be a major mechanism in steady state (83), it has been demonstrated to be important under inflammatory conditions, for example, in cancer (84). An additional factor that has been revealed to induce neutrophil apoptosis is TNFα (85). Notably, type I IFN signaling has been shown to increase expression of TNFα by neutrophils (8). Decreased neutrophil apoptosis in the absence of endogenous type I IFNs could be also due to the decreased production of cytotoxic ROS by TANs (34).

## Type I IFNs Influence Neutrophil Migration in Tumor-Bearing Mice

The process of neutrophil migration to the site of inflammation depends on the several ligand–receptor interactions, including chemokine sensing and sensing of activated endothelium in inflammatory site. Retention of immature neutrophils in BM is due to high expression of CXCR4 on the cell surface and its interaction with CXCL12 secreted by stromal cells. Attenuation of CXCR4 signaling and upregulation of CXCR2 on neutrophils is an important mechanism by which these cells are mobilized into the circulation under inflammatory conditions (71). Subsequent neutrophil migration to tissues is determined by interacting of surface chemokine receptors and chemokines forming concentration gradient. Mature neutrophils are characterized by the high expression of CXCR2 (86, 87). The ligands of this receptor (CXCL8 in humans and CXCL1, CXCL2 in mice) are responsible for homing of mature neutrophils into tissues. Lungs, liver, and spleen are the major producers of CXCR2 ligands under normal conditions and conclude considerable neutrophil marginated pool in microvascular bed (88). It is suggested that the high expression of CXCR2, CXCR4, and CCR7 ligands in lungs, liver, BM, and lymph nodes is one of the reasons responsible for metastases homing toward these organs in certain types of cancer (89, 90).

Tumor tissue seems to be a significant source of chemokine ligands of CXCR2 (91–93) and forms chemokine gradient attracting neutrophils. Low CXCL1 or CXCL2 level in BM and high level of these chemokines in the tumor form gradient in tumor-bearing mice, thus attracting neutrophils into tumor site. Correspondingly, the expression of CXCR2 is the highest on neutrophils from BM and blood, and is downregulated after reaching the tumor (69). Of note, the migration of neutrophils is downregulated by endogenous type I IFNs *via* suppression of chemokines. Expression of CXCL1 and CXCL2 in blood and tumor was significantly higher in *Ifnb1^−/−^* mice as compared to wild-type (WT) controls. On the other hand, the expression of CXCL5, which is known to compete with CXCL1 and CXCL2 for CXCR2-binding site, was upregulated in blood of WT mice. This could be responsible for the inhibited migration of neutrophils into tumor tissue in WT animals, since they are trapped in the blood due to high concentration of CXCL5. Treatment of tumor-bearing IFN-deficient mice with recombinant murine IFN-β downregulated CXCL1 and CXCL2 expression in blood and tumor to the levels observed in control mice (69).

An additional chemokine/receptor axis involved in migration of neutrophils into tumor site is CXCL12/CXCR4 axis. This axis has also been shown to be downregulated by type I IFNs. Endogenous type I IFNs inhibit CXCR4 expression on neutrophils and block CXCL12 expression in tumors leading to suppressed migration of neutrophils toward the tumor (69).

Rolling, adhesion, and migration of neutrophils to the site of inflammation are mediated by the interaction of endothelial adhesion molecules and their ligands on leukocytes (94). Mature and activated neutrophils are characterized with decreased surface expression of l-selectin CD62L (95). Importantly, in the absence of endogenous type I IFNs tumor-bearing mice show significantly increased percentage of CD62L^+^ circulating neutrophils (8), which could result in increased migration to tumor site. All described mechanisms explain the increased migration of neutrophils into tumors leading to the enhanced tumor growth in IFN-deficient mice.

## Regulation of Oxidative Burst by Type I IFNs

Type I IFNs were shown to regulate the most prominent antitumor feature of neutrophils, i.e., their ability to directly kill tumor cells (8). Neutrophil cytotoxicity includes both direct and antibody-dependent cell-mediated cytotoxicity (*via* recognition of opsonized cells). Functional activity of neutrophils is determined by large spectrum of secretory granules and vesicles rather than production of proteins *de novo*. The primary azurophilic granules containing myeloperoxidase and other acid hydrolases, as well as neutral proteases (cathepsin G, elastases, and collagenases), are responsible for pathogen degradation. Secondary (specific) granules are large stores for soluble mediators as well as for NADPH oxidase that supports oxidative burst. The tertiary granules and secretory vesicles support migration and interaction of neutrophils with the environment (96).

Cytotoxicity depends on developmental stage of the cell (63) but also on the microenvironment. In animal models, TANs show reduction of cytotoxicity in comparison to blood-derived neutrophils, indicating further influence of the tumor milieu on neutrophil activation and function (8). Type I IFN signaling is essential for neutrophils to facilitate some of their functions (63). Accordingly, decreased spontaneous production of cytotoxic ROS by tumor-infiltrating neutrophils was demonstrated in mouse models deficient in endogenous IFNs (34), which was linked to significantly reduced cytotoxicity of tumor neutrophils in such mice, compared to IFN-sufficient animals. Treatment of *Ifnb1^−/−^* mice with recombinant IFN-β-restored neutrophil cytotoxicity (8).

## Decreased Neutrophil Extracellular Trap Formation by Neutrophils Deficient in Endogenous Type I IFNs

Neutrophil extracellular traps consist of nuclear or mitochondrial-derived web-like DNA strands released from neutrophils that are equipped with histones and bactericidal proteins. The process of NETs release is called NETosis and it is an unique form of cell death. NETosis is a mechanism of distinct killing of extracellular pathogens with high local concentration of effector components (97). The intracellular components shifted extracellularly become a target for macrophages, which destroy attached pathogen as well (98). During this process, neutrophils kill extracellular pathogens while minimizing damage to the host cells. Tumor environment obviously and strongly activates neutrophils and initiates NETs release (8). There are conflicting data about the role of NETs formation during tumorigenesis. On the one hand, it is postulated that released NETs improve efficient tumor cell killing by neutrophils (99). On the other hand, there are studies suggesting NETs as a mechanism supporting metastasis formation (100). One could speculate that the subsequent fate of trapped tumor cells depends on the activation of the neutrophils and their ROS release.

Interferons seem to influence the process of NETosis. Priming with IFN-α or IFN-γ with subsequent C5a activation triggers release of NETs in mature human neutrophils. Notably, immature neutrophils are not able to release NETs in this condition, probably due to the lack of IFN signaling pathway mediators (63). Another animal model study revealed that blood neutrophils isolated from type I IFN-deficient tumor-bearing mice display significantly lower NETs formation capacity compared to WT controls. This is accompanied by less efficient tumor cell killing by these cells and was in agreement with observed enhanced tumor growth in such mice (8).

## Suppression of Pro-Angiogenic Properties of Neutrophils by Type I IFNs

Effective angiogenesis is essential for successful tumor growth. One of the developmental hallmarks of a tumor is the induction of angiogenesis, i.e., the formation of new blood vessels. Small tumors up to a size of 1–2 mm^3^ can be supplied with oxygen and nutrients by the surrounding tissue. For larger tumors, this is no longer sufficient. The tumor has to alter its angiogenic phenotype and the so-called angiogenic switch – the induction and assembly of tumor vasculature – has to take place (101, 102). Interestingly, myeloid cells like neutrophils are known to take part in tumor vascularization since they are known to be the source of the variety of pro-angiogenic factors, including VEGF and angiogenic chemokines (103). MMPs and other enzymes released by neutrophils provide degradation of extracellular matrix and facilitate vessel growth (104).

The role of type I IFNs in inhibition of tumor angiogenesis has been suggested before (105, 106). Recent data show also the significant impact of TANs on angiogenic processes in tumor and the important role of type I IFNs in the modulation of these processes (18). In *Ifnb1^−/−^* mice, considerably higher tumor growth was observed, accompanied by boosted angiogenic processes. Enhanced content of fully developed functioning vessels, completely covered by pericytes, was found. Moreover, the number, area and the perimeter of vessels in tumors of such mice were significantly higher than in WT mice. Notably, these tumors were strongly infiltrated by neutrophils that were found in close vicinity of vessels. Neutrophils isolated from *Ifnb1^−/−^* mice show significantly higher expression of VEGF and MMP9 (18). Moreover, CXCR4 was upregulated on these cells. CXCR4, is known to be overexpressed in highly vascularized tumors (107, 108), and its ligand CXCL12 is apparently induced under hypoxic conditions in accordance with the triggering of the angiogenic switch. Depletion of neutrophils in this model led to reduction of number of developed vessels and subsequent retardation of tumor growth as compared to untreated animals (18). *In vitro* treatment of TANs isolated from *Ifnb1^−/−^* mice with low levels of IFN-β restored expression of pro-angiogenic factors to control levels (18).

Certain chemokines (CXCL1, CXCL2, CXCL3, CXCL5, CXCL6, and CXCL8) are known to mediate angiogenic processes through direct activation of endothelial cells *via* CXCR2 receptor or recruit pro-angiogenic immune cells and endothelial progenitors to the neovascular niche (109). Type I IFNs were shown to decrease production of some of these chemokines, which could serve as an additional antiangiogenic mechanism during tumorigenesis (69).

Reactive oxygen species are also considered to be regulators of endothelial cell functions. While high amounts of ROS are toxic for endothelial cells and reveal antiangiogenic properties, low concentrations of NO and H_2_O_2_ can serve as intracellular mediators of signal transduction to stimulate vascular smooth muscle cells to support angiogenesis [reviewed by Irani (110)]. Thereby, increased IFN-dependent ROS production by tumor-infiltrating neutrophils (34) can additionally exert an antiangiogenic effect.

## The Regulation of Adaptive Immune Responses by Neutrophils Stimulated with Type I IFNs

Accumulating data suggest that neutrophils may influence adaptive immunity by acting either indirectly (*via* APCs) or directly on T cells. T cells are considered to be key players involved in antitumor immunity; yet, many other components of the immune system take part in this process. Antigen presentation is an important link between innate and adaptive immune responses. Two main mechanisms are involved in this process. Fragments of intracellular pathogens are presented on MHC class I complex to CD8^+^ cytotoxic T cells. Extracellular pathogens, after phagocytosis and procession, are bound in phagolysosome with MHC class II molecules and are presented to CD4^+^ T-cells (111). The third mechanism of antigen presentation, called “cross-presentation,” shares features of previous two. In this process, APCs translocate extracellular antigen from the endocytic vesicle to the cytosol and present it on MHC class I to CD8^+^ T cells (cross-priming) (112). In the last two cases, appropriate activation of APCs is necessary to induce effective immune response. In the absence of activating stimuli or in anti-inflammatory environment, APCs stimulate abortive T-cell responses, which lead to tolerance (113).

All neutrophils constitutively express MHC class I. Murine neutrophils, both circulating and resident, are known to express MHC class II and can potentially play a role in antigen presentation together with macrophages and DCs. To the contrary, human circulating neutrophils do not express MHC class II under normal conditions, but there is an evidence of antigen-presenting function of these cells in certain inflammatory conditions, including autoimmune diseases (114, 115) as well as after treatment with GM-CSF (116) or IFN-γ (117). In patients with cancer, no MHC class II expression on circulating neutrophils was observed (33), arising a question about TANs participating in antigen presentation. Immature neutrophils that are released from BM as a result of tumor-driven emergency myelopoiesis were shown to become activated with cytokines released in tumor microenvironment (GM-CSF, IL-4, TNF-α) and acquire molecular features characteristic for DCs. Such activated DC-like cells express DC-associated surface molecules cluster of differentiation CD1a, CD1b, CD1c, MHC class II, and costimulatory molecules CD80, CD86, CD40, as well as ICAM-1 and CD5. At the same time, these cells downregulate CD15 and CD65 expression. Altogether, this leads to effective presentation of antigen to CD4^+^ T cells, thus activating antitumor immune responses (118). Recently, Eruslanov et al. demonstrated that neutrophils augment T cell proliferation in a positive-feedback loop *via* upregulation of ICAM-1 and costimulatory molecules like CD86, OX40L, and 4-1BBL on the neutrophil surface (32). ICAM-1 was also shown to act as a costimulatory molecule taking part in antigen presentation to T cells and is crucial for T cell activation under conditions where costimulation by CD80 and CD86 is low (119). High ICAM-1 expression can, therefore, induce the activation of cytotoxic CD8^+^ T cells (120) as well as repress the secretion of immunosuppressive IL-10 by CD4^+^ T cells (121). Mature neutrophils upregulate ICAM-1 (122) and, therefore, can participate in antigen presentation. Importantly, it was recently demonstrated that type I IFNs strongly upregulate ICAM-1 expression on neutrophils (8).

Type I IFNs support systemic immunity against tumor targets by upregulation of MHC class I expression as well as enhancement of cytotoxic T cell responses and activation of NK cells (60). They are also known to induce MHC class II expression on monocytes (123) as well as to induce cross-priming of CD8^+^ cells against exogenous antigens (112).

Thus, neutrophils are able to influence adaptive immune responses, either by directly presenting peptide–MHC class I complexes, MHC class II complexes, or by delivering peptides to other APCs for presentation. Cross-presentation by these cells occurs actually earlier than in professional APCs (31). Possibly, neutrophils may directly present peptide to effector T cells *in vivo*, inducing cytokine production, whereas DCs after receiving neutrophil-derived antigenic peptides may migrate to lymphoid organs to initiate T cell responses (124).

Apart from activating T cells *via* antigen presentation, neutrophils could attract T cells to the sites of inflammation, e.g., growing tumors. CD8^+^ T cells are attracted to the inflamed tissue *via* CXCL12 (125). Notably, this chemokine was shown to be produced by TANs and downregulated by type I IFNs (18). Moreover, neutrophils produce cytokines-stimulating T cell differentiation and activation, e.g., IFN-γ or TNF-α. Leschner et al. could show that expression of TNF-α is strongly enhanced in blood and tumors of tumor-bearing mice (126). Importantly, type I IFNs upregulate TNF-α expression in TANs (8), thus regulating lymphocyte antitumor responses. On the other hand, peripheral blood neutrophils, under specific conditions, e.g., late stage of tumor, can also suppress antigen non-specific T cell proliferation through the release of arginase-1, TGF-β, and the production of ROS (127–129). Expression of ROS was also shown to be stimulated by type I IFNs (8, 34), once again demonstrating the strong involvement of this cytokine family in the activation of adaptive immune responses leading to the restriction of tumor growth.

## The Efficiency of Metastatic Spread Depends on Neutrophils and is Inhibited by Type I IFNs

Metastases are associated with unfavorable prognosis in cancer (130). Metastatic spread is a complex process that includes cells shedding from a primary tumor, their migration in circulation, extravasation, and initiation of secondary tumor growth. Recently, it was postulated that metastases from primary tumors do not migrate and home undirected into sites of secondary growth, but are guided by cells that form the so-called pre-metastatic niche (19, 29, 131). Major component of the pre-metastatic niche are neutrophils. They are apparently controlled by factors produced by the primary tumor and are responsible for the preference of metastasizing tumor cells to certain organs. Different neutrophil-mediated mechanisms of metastatic spread are described, including promotion of tumor cell extravasation by binding ICAM-1 on tumor cells (132) or by catching tumor cells *via* NETs (100).

Endogenous type I IFNs play essential role in modulating neutrophil functions in context of metastatic processes. In mice lacking endogenous type I IFNs, higher metastatic load was observed in the lung as compared to WT animal, which was accompanied by strong neutrophil accumulation in this organ (18, 20). One of the reasons attributed to this phenomenon was the elevated plasma level of G-CSF and increased expression of CXCR2 on neutrophils (20). In the absence of endogenous type I IFN signaling, neutrophils express more CXCR2 and are capable to extravasate more actively to the organs producing high levels of CXCL1 or CXCL2. Such organs that are predisposed to metastasis formation are lungs, liver, and spleen, as mentioned previously. Neutrophils accumulating in pre-metastatic lungs support tumor cell extravasation and proliferation by release of pro-metastatic proteins, e.g., Bv8, MMP9, S100A8, and S100A9. S100A8 and S100A9 are known to influence tumor cell proliferation, survival, and migration (133) as well as to stimulate migration and proliferation of neutrophils themselves. Bv8 induces tumor cell extravasation (134) and increases neutrophil accumulation within pre-metastatic tissue. MMP9 is responsible for formation of leaky vasculature in the pre-metastatic lung (131) and supports tumor cells survival in this organ. The expression of all above factors is significantly enhanced in type I IFN-deficient mice and is suppressed by the recombinant IFN treatment (18, 20). Notably, G-CSF, that is also downregulated by type I IFNs, is known to enhance expression of Bv8, S100A8, S100A9, and MMP9 in neutrophils and thus might also be directly involved in regulating pre-metastatic niche formation (134). Neutrophils from IFN-deficient mice show also reduced cytotoxicity against tumor cells leading to enhanced metastasis in such mice (20). Moreover, Bidwell et al. demonstrated that, in a mouse model, early initiated administration of recombinant type I IFNs leads to reduced bone metastases and prolonged survival of the host (135). This indicates that endogenous type I IFNs effectively suppress the formation of pre-metastatic niche on multiple levels.

## Clinical Aspects of Type I IFN-Mediated Polarization of Neutrophils

The efficacy of type I IFN therapy for various malignancies has been investigated for many years. IFN therapy has been clinically evaluated as the treatment of melanoma (136, 137), renal cell carcinoma (138, 139), myeloproliferative disorders (140, 141), lymphomas (142), neuroendocrine tumors (143) as well as vascular neoplasias including pulmonary hemangiomatosis (144), infantile hemangiomas (145), Kaposi’s sarcoma (146), and malignant hemangiopericytomas (147).

Lately, the role of type I IFNs in modulation of immune cell activation in tumor context is getting attention. It is generally accepted that immune cells play important role in the regulation of tumor growth. Neutrophils, both circulating and tumor associated, represent an independent prognostic marker in a broad variety of neoplasias (148, 149); therefore, several studies aimed to modulate the immune system in order to suppress pro-tumoral components and enhance antitumoral immune responses. This has determined the increasing interest in type I IFN treatment. The evidence that IFNs play a role in neutrophil polarization was supported with clinical observations. Recently, the increase of ICAM-1 expression on the neutrophils isolated from melanoma patients undergoing adjuvant type I IFN therapy was shown (8). Notably, the treatment was associated with reduced migratory capacity of neutrophils in such patients. Blood-derived neutrophils from melanoma patients upon adjuvant type I IFN treatment significantly downregulate their IL-8 receptor (CXCR1 and CXCR2) expression. It affects neutrophil migration from the BM and is of high clinical importance due to poor prognosis for tumor patients with elevated neutrophil numbers in blood and tumor. Notably, neutrophil amounts in type I IFN treated patients were lower, compared to untreated patients (8).

Immunotherapy with alpha IFNs is used for patients with different types of malignancies. Nevertheless, its efficacy is limited and only a small proportion of patients benefit from such treatment. Notably, the level of responsiveness to IFN treatment varies among individuals. This might be due to genetic polymorphism in type I IFN-related genes that have been shown to exert a significant impact on survival and therapy outcome in melanoma patients (61). Importantly, humans with impaired type I IFN signaling, due to STAT2 deficiency, have been identified (62). Another reason for impaired therapy response could be a suppression of pathways involved in IFN signal transduction in different microenvironment conditions, e.g., in GM-CSF presence (63). One of the factors reflecting the sensitivity of neutrophils to IFN-α therapy is a study by Azuma et al. showing a favorable survival predictive response correlated with a decrease in the number of circulating neutrophils after IFN-α treatment in patients with metastatic renal cell carcinoma (150).

## Concluding Remarks

**Figure 1:**
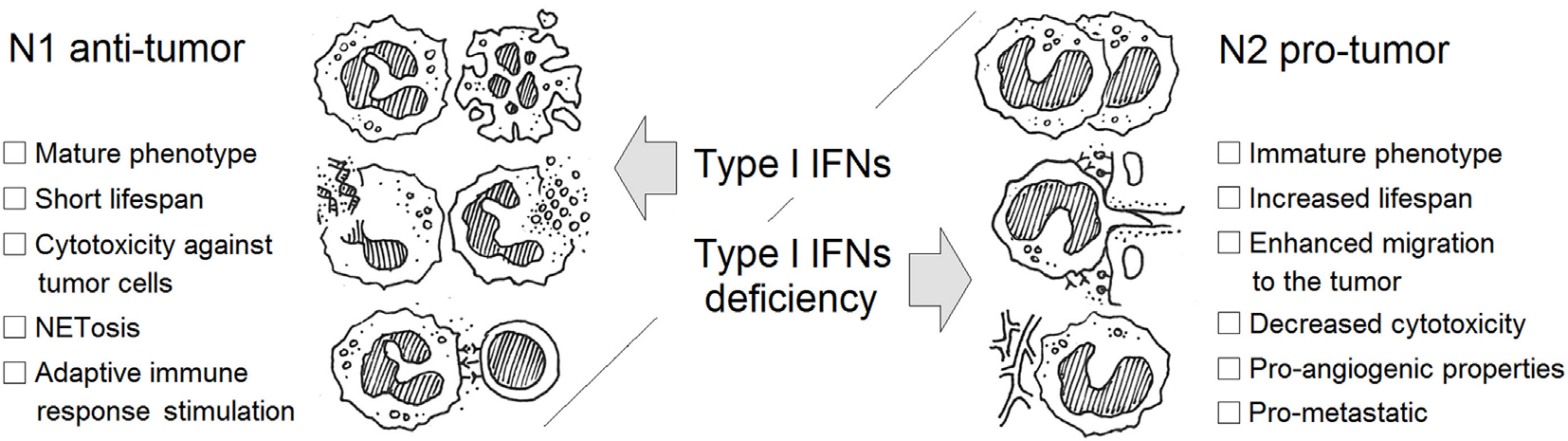
**Type I interferon (IFN)-dependent regulation of neutrophil polarization in cancer**. Neutrophils may be divided into N1 antitumor and N2 pro-tumor cells in tumor situation. The activation and differentiation of these cells during tumorigenesis is determined by the growth factor and cytokine milieu in the tumor. Type I IFNs are potent drivers of the transition into antitumor N1 phenotype.

Type I IFNs are one of few cytokines known to alter polarization of neutrophils in tumor-bearing hosts. IFNs drive neutrophils to an antitumor and antimetastatic phenotype in numerous ways: due to restriction of neutrophil survival and migration to tumor site, *via* enhancement of neutrophil cytotoxicity and NETs formation, suppression of pro-angiogenic properties of neutrophils, and inhibition of the pre-metastatic niche formation by these cells (Figure 1; Table 1). Importantly, IFNs need initial trigger, such as inflammation accompanying tumor growth, to exert their neutrophil polarizing effect. In healthy, tumor-free mice, no alteration in neutrophil activation and polarization due to IFNs could be observed. Inflammation and cytokine milieu in tumor, together with functional type I IFN signaling, are responsible for subsequent alteration of neutrophil activation leading to modifications of their phenotype into tumor inhibiting. In the situation when IFN signaling is disturbed, neutrophils are polarized into pro-tumor phenotype and effectively support tumor growth. Thus, the vicious circle enhancing tumor progression and metastasis is formed. In this situation, restoring the pool of type I IFNs by using exogenous treatment should modulate neutrophilic phenotype providing therapeutic option to overcome neutrophil-mediated immunosuppression thus leading to the restriction of tumor growth.

**Table 1:** **Type I IFN-dependent regulation of neutrophil polarization in cancer**.

	Sufficient type I IFN signaling	Impaired type I IFN signaling
**Polarization of neutrophils**	**N1 anti-tumor**	**N2 pro-tumor**
**THE TURNOVER AND THE LIFESPAN OF NEUTROPHILS**		
**Neutrophil expansion in bloodstream and tumor**	↓	↑
Expression of G-CSF by neutrophils	↑	↓
Expression of pro-apoptotic factors by neutrophils (caspase 3, TNFα, Fas, ROS production)	↑	↓
Expression of antiapoptotic factors by neutrophils (Bcl-XL)	↓	↑
**MIGRATION OF NEUTROPHILS TO THE TUMOR SITE**		
**Neutrophil expansion in bloodstream and tumor**	↓	↑
CXCR2 – CXCL1, CXCL2 axis activation	↓	↑
CXCR4 – CXCL12 axis activation	↓	↑
CD62L expression on circulating neutrophils	↓	↑
**KILLING OF TUMOR CELLS**		
**Neutrophil cytotoxicity against tumor cells**	↑	↓
ROS production by TAN	↑	↓
Neutrophil extracellular traps formation	↑	↓
**REGULATION OF ADAPTIVE IMMUNE RESPONSE**		
Expression of co-stimulatory molecules (ICAM-1)	↑	↓
Expression of cytokines (TNFα)	↑	↓
**ANGIOGENESIS AT THE TUMOR SITE**		
**The number of fully developed vessels in the tumor**	↓	↑
Expression of VEGF, MMP9 by TAN	↓	↑
CXCR2 – CXCL1, CXCL2 axis activation	↓	↑
CXCR4 – CXCL12 axis activation	↓	↑
ROS production by TAN	↑	↓
**FORMATION OF THE PRE-METASTATIC NICHE**		
**Metastatic load in organs**	↓	↑
Accumulation of neutrophils in metastatic organs	↓	↑
Expression of pro-metastatic proteins (Bv8, S100, and MMP9)	↓	↑
CXCR2 – CXCL1, CXCL2 axis activation	↓	↑

*Neutrophils may be biased into N1 antitumor and N2 pro-tumor cells in tumor situation. The activation and differentiation of these cells during tumorigenesis is determined by the growth factor and cytokine milieu in the tumor. Type I interferons are potent drivers of the transition into antitumor N1 phenotype*.

*GCSF, granulocyte colony-stimulating factor; TNFα, tumor necrosis factor alpha; ROS, reactive oxygen species; TAN, tumor-associated neutrophils; VEGF, vascular endothelial growth factor; MMP9, matrix metallopeptidase 9*.

## Author Contributions

EP: drafting of the manuscript, writing of the manuscript, final approval of the submitted version, agreed to be accountable for all aspects of the work. SL: writing of the manuscript, final approval of the submitted version, agreed to be accountable for all aspects of the work. JJ: concept and design of the manuscript, drafting and writing of the manuscript, final approval of the submitted version, agreed to be accountable for all aspects of the work, submission of the manuscript, and corresponding author.

## Conflict of Interest Statement

The authors declare that the research was conducted in the absence of any commercial or financial relationships that could be construed as a potential conflict of interest. The reviewer PS and handling Editor declared their shared affiliation, and the handling Editor states that the process nevertheless met the standards of a fair and objective review.
